# Estimating Trans-Seasonal Variability in Water Column Biomass for a Highly Migratory, Deep Diving Predator

**DOI:** 10.1371/journal.pone.0113171

**Published:** 2014-11-26

**Authors:** Malcolm D. O'Toole, Mary-Anne Lea, Christophe Guinet, Mark A. Hindell

**Affiliations:** 1 Institute of Marine and Antarctic Studies, University of Tasmania, Hobart, Australia; 2 Marine Predator Department, Centre détudes biologiques de Chizé, Villiers-en-Bois, France; Pacific Northwest National Laboratory, United States of America

## Abstract

The deployment of animal-borne electronic tags is revolutionizing our understanding of how pelagic species respond to their environment by providing *in situ* oceanographic information such as temperature, salinity, and light measurements. These tags, deployed on pelagic animals, provide data that can be used to study the ecological context of their foraging behaviour and surrounding environment. Satellite-derived measures of ocean colour reveal temporal and spatial variability of surface chlorophyll-a (a useful proxy for phytoplankton distribution). However, this information can be patchy in space and time resulting in poor correspondence with marine animal behaviour. Alternatively, light data collected by animal-borne tag sensors can be used to estimate chlorophyll-a distribution. Here, we use light level and depth data to generate a phytoplankton index that matches daily seal movements. Time-depth-light recorders (TDLRs) were deployed on 89 southern elephant seals (*Mirounga leonina*) over a period of 6 years (1999–2005). TDLR data were used to calculate integrated light attenuation of the top 250 m of the water column (*LA_250_*), which provided an index of phytoplankton density at the daily scale that was concurrent with the movement and behaviour of seals throughout their entire foraging trip. These index values were consistent with typical seasonal *chl-a* patterns as measured from 8-daySea-viewing Wide Field-of-view Sensor (SeaWiFs) images. The availability of data recorded by the TDLRs was far greater than concurrent remotely sensed *chl-a* at higher latitudes and during winter months. Improving the spatial and temporal availability of phytoplankton information concurrent with animal behaviour has ecological implications for understanding the movement of deep diving predators in relation to lower trophic levels in the Southern Ocean. Light attenuation profiles recorded by animal-borne electronic tags can be used more broadly and routinely to estimate lower trophic distribution at sea in relation to deep diving predator foraging behaviour.

## Introduction

Chlorophyll-a is an important biological parameter in the Southern Ocean and is considered a useful indicator of spatial and temporal variability of primary productivity [1]–[3]. To understand the foraging behaviour and habitat utilisation of higher trophic organisms requires knowledge of lower trophic dynamics, coupled with information on how organisms respond to these changes. Indeed, satellite measurements of ocean colour have revealed the complex temporal and spatial variability of weighted average near-surface chlorophyll-a concentration [4], but the quantity and quality of information obtained in this way is affected by cloud cover. Consequently, information from high latitudes and during the winter months is often sparse [5], [6] and correspond poorly with marine animal behaviour. Moreover, to improve data availability, these patchy satellite data are often merged at spatio-temporal scales not necessarily relevant to marine animal behaviour. While fluorometers and water samples from ship-based surveys are the only in-vivo and in-vitro measurements to determine chlorophyll-a concentration, it is both costly and logistically difficult if collecting simultaneously with animal behaviour. In recent years, additional ocean data recorded by animal-borne electronic tags have been used to supplement other data from buoys and satellites (e.g., [7], [8]) and have improved our understanding of the relationship between marine predator distribution and environmental parameters, including chlorophyll-a [9], [10]. Indeed, miniaturised fluorometers have now been deployed, in some instances simultaneously with light sensors, on elephant seals to estimate chlorophyll-a in the water column [11], [12] but are costly and available data are scarce. Therefore, understanding lower trophic variability (i.e. phytoplankton) and its influence on marine predators in the Southern Ocean is still hampered by a lack of concurrent data.

Time-depth-light recorders (TDLRs) provide detailed information on dive behaviour of a wide range of animals over extensive areas [13], [14], and are often coupled with sensors that record environmental data (*e.g.* temperature and salinity). Southern elephant seals (*Mirounga leonina*) are ideal platforms for these oceanographic sensors due to their circumpolar distribution extensive foraging across the Southern Ocean [9]. They are also a deep diving animals, diving up to 2000 m [15] while performing on average 60 dives per day (Hindell et al. 1991). Elephant seals can be used to measure *in situ* environmental conditions and provide important habitat information for the seals [9]. Seals equipped with sensors that collect information such as temperature, salinity can cover areas not sampled by conventional techniques (e.g. ship-based survey, satellite images), including within the sea-ice zone (*i.e.* south of 60°S) where it is particularly difficult to sample physical parameters of the ocean [7]. Furthermore, post-moult elephant seals are also at sea throughout winter when data collected by conventional techniques is scarce.

Light levels recorded by animal-borne sensors are commonly used to infer day length as a means of estimating geographical position [16], [17], and can also be used as means of recording light levels at depth during animal diving [18]–[20]. Experiments have demonstrated the concept of estimating chlorophyll-a distribution from light-depth data compared to fluorescence (e.g., [10], [12]). Fluorometers estimate chlorophyll-a by measuring its fluorescence intensity. Light sensors instead measures ambient light, which is attenuated throughout the water column for two reasons: (1) physical properties of the seawater and (2) quantity of inorganic and organic particles suspended (or dissolved) in the water column [21]. The Southern Ocean is typically characterised by Case I waters, whereby phytoplankton comprises the main source of particles suspended within the euphotic zone [21], [22], and is consequently the main cause of light attenuation if we assume related coloured dissolved organic matter (CDOM) and detritus degradation products covary with phytoplankton [23] and physical properties are constant [24]. Indeed, it was Smith and Baker [1] that introduced the concept of measuring the bio-optical properties of the water column to estimate the concentration of chlorophyll-a in the ocean. A study by Teo et al. [10], one of the first to use light levels collected by Pacific blue fin tuna (*Thunnus orientalis*) to estimate chlorophyll-a distribution, found a positive relationship between light attenuation at depth and *in situ* chlorophyll-a collected by both water samples and fluorometers. Light levels collected by elephant seals equipped with light sensors were also strongly correlated with concurrent *in situ* fluorometer data [12]. Despite their findings, these studies were not performed over multiple seasons; instead tested over a much shorter time scale. Nor was light attenuation compared with satellite-derived chlorophyll-a estimates (hereafter *chl-a*). More recently, Guinet et al. [11] used a multi-seasonal dataset over several years and found *chl-a* to be related to surface chlorophyll-a estimates from seal-borne fluorometers. The bio-optical relationship between chlorophyll-a and phytoplankton and does vary according to phytoplankton taxonomic composition [25] but are still considered to correlate well with each other. To our knowledge, no study has used light level and depth data to generate a phytoplankton index that matches daily seal movements while at sea.

This study examined the feasibility of using light collected from TDLRs to calculate an index of phytoplankton distribution that is concurrent with marine animal behaviour in the Southern Ocean. We also highlight the advantages of a phytoplankton index recorded simultaneously with the foraging behaviour of a top marine predator, particularly at times of the year where *chl-a* data is lacking. Analyses were performed in Case 1 waters over multiple seasons between 1999 and 2005. Our primary objectives included:

- providing an index of phytoplankton density at the daily scale that is concurrent with the movement and behaviour of seals throughout their entire foraging trip;
- demonstrating that our index is consistent with typical seasonal chl-a patterns;
- examining the efficacy of using a light-based index to estimate phytoplankton distribution.

## Materials and Methods

### Ethics statement

All necessary permits were obtained for the described field studies. Elephant seal research was sanctioned by the University of Tasmania Animal Ethics Committee (permit A6738) and the Australian Antarctic Science Advisory Council Ethics Committee (project 2794). Permits and permission to carry out research on Macquarie Island was obtained from Parks and Wildlife Service Tasmania.

TDLRs (Mk6, Mk7, Mk8 and Mk9; Wildlife Computers, Redmond, WA, USA) were attached to both post-breeding and post-moult adult female southern elephant seals (n = 89) at Macquarie Island (54°35′S, 158°58′E, Table 1) from 1999 to 2005. The seals were approached by foot and temporarily restrained with a head bag and anaesthetised intravenously with a 1∶1 mixture of tiletamine and zolazepam (0.5 mg kg^−1^) [26], [27]. TDLRs were attached to the pelage above the shoulders using a two component industrial epoxy (Araldite AW 2101) [28]. Seals were observed during recovery from anaesthesia and allowed to enter the water when no longer sedated. TDLRs were retrieved at the end of the foraging trip once the seal had hauled out on land by repeating the above restraint procedures. These tracking devices or attachment method did not adversely affect individual performance and fitness over the short (seal growth) or long (seal survival) term [29].

**Table 1:** Summary of tag deployments for 89 female elephant seals: year, trip, tag type used, number of individuals tagged, and period of data records (start to finish dates).

				Period of data records	
Year	Trip	Tag type	No. individuals	Start	Finish
1999	PB	mk6	4	23-Oct-1999	12-Jan-2000
1999	PB	mk7	15	15-Oct-1999	17-Jun-2000
2000	PM	mk7	9	26-Jan-2000	15-Oct-2000
2000	PB	mk7	11	21-Oct-2000	14-Jun-2001
2001	PM	mk7	3	9-Feb-2001	3-Oct-2001
2001	PM	mk8	3	15-Jan-2001	13-Oct-2001
2002	PM	mk7	3	30-Jan-2002	22-Sep-2002
2002	PM	mk8	12	26-Jul-2001	7-Nov-2002
2004	PM	mk8	12	30-Jan-2004	19-Oct-2004
2004	PB	mk8	7	18-Oct-2004	31-Jan-2005
2004	PM	mk9	3	24-Jan-2004	15-Dec-2004
2004	PB	mk9	2	22-Oct-2004	5-May-2005
2005	PM	mk8	3	11-Jan-2005	13-Oct-2005
2005	PM	mk9	2	19-Jan-2005	15-Oct-2005

*PB* - post-breeding, *PM* - post-moult.

### Tag data

TDLRs measured time, depth and light at 30 s intervals for the duration of each foraging trip. Mk6–Mk8 tags used uncorrected watch crystals to measure time. They were offset to spread the time error (TE) over the likely range of seawater temperatures (T) (TE = (1×10^−5^−3.5×10^−8^×(T−25)^2^)×10^6^ µs). Mk9 tags used a temperature correction algorithm to keep the time error within 1 ppm. Depth measurements were made by a pressure transducer calibrated by the manufacturer (±6 m). Light values are converted on-board the logger via a log treatment (see Figure S1) to compress the light measurements to a three digit value, thereby giving a linear relationship and increase the resolution at lower light levels. The light sensor data can be used to identify dawn/dusk events down to 300 m in clear waters and is temperature-compensated for the entire light level range (Wildlife Computers). The wavelength at the centre of the light sensor parabolic-shaped pass-band filter is ∼430 nm and consequently the sensor only reads the violet/blue light band (370 nm–470 nm). All other bands of light are rejected and not measured. The light sensor measures on a scale of 20 readings per decade, so the light level error is considered to be 1/20^th^ of a decade. Tags also recorded temperature (±0.1°C). The lag in temperature measurement (inherent in the design of the TDLRs) was accounted for (see [30], [31]).

### Data extraction

Twice daily at-sea location estimates were derived from the recorded light levels for sunrise and sunset using the geo-location procedure outlined inThums et al. [32]. Geo-location by light enables animal movement estimation, based on measurements of light intensity over time recorded by the in-built light sensor of each TDLR [17]. However, an inherent problem with this approach is that an array of factors may change the natural light intensity pattern, thereby affecting the accuracy and precision of location estimates calculated from these light patterns [33]–[35]. With the incorporation of the ‘tripEstimation’ method (see [36]) geo-location mean longitudinal and latitudinal error is shown to be estimated at ∼57±9 km (i.e. 0.83°) and ∼54±8 km (i.e. 0.49°) respectively (Chew unpublished). All dive recorders were corrected for drift in the pressure sensor using a customised zero-offset correction routine. We then identified individual dive cycles, defined as the first sub-surface record until the last surface interval of the subsequent post-dive surface interval below 10 m. The surface interval encompassed depth values between 0 and 10 m. This tolerance accounted for subsurface movements of seals between dives.

### Environmental data

#### Satellite-derived chlorophyll-a estimates

The *chl-a* data (mg m^−3^) was estimated from Sea-viewing Wide Field-of-view Sensor (SeaWiFs) images [37]. Because of the patchy nature of these data at high latitudes, particularly during winter, we used 8-day *chl-a* composites at 0.1° resolution (http://oceancolor.gsfc.nasa.gov/).

#### Sea ice

We extracted sea ice data from daily satellite images (grid cell size of 25 km×25 km) [38]. Satellite *chl-a* data in regions with >20% sea ice coverage were excluded from analyses as reflective irradiance from the ice may affect the accuracy of satellite imagery (P. Strutton, Personal Communication). Sea ice data were also used to calculate the seasonal mean sea ice extent between 1999 and 2005. The sea ice extent was defined by the open ocean (i.e. ice-free pelagic region) – sea ice (>50% concentration) interface.

#### Bathymetry

We aggregated bathymetry data, derived from the ETOPO2 bathymetry data set at 2′ resolution (http://www.ngdc.noaa.gov/mgg/global/global-.html), to calculate mean bathymetric depth of each 1°×1° grid cell associated with each seal location.

#### Mixed layer

In order to assess changes to phytoplankton density using integrated light attenuation we consider total phytoplankton in the water column, the bulk of which is found within the mixed layer [39]. Temperature and profiles recorded by the TDLRs were used to identify the mixed layer depth (hereafter MLD) for each dive to establish the vertical extent of phytoplankton in the water column. A custom broken stick method was used to find the greatest inflection point along each temperature-depth profile to a depth of 350 m (limit of light sensor sensitivity is ∼300 m). The inflection point was considered the MLD if the difference between temperature at the surface (∼10 m) and temperature inflection point was greater than 0.2°C [40].

**Figure 1:**
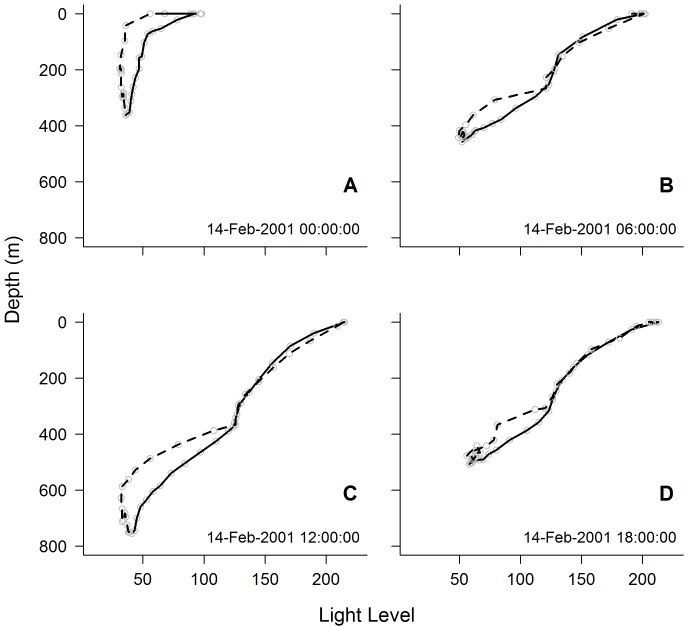
Examples of light-depth profiles collected from the descent (solid) and ascent (dashed) phases of dives. Profiles recorded at local (A) midnight, (B) 6am, (C) noon and (D) 6pm on 14 February 2001. Light level values are related to blue light intensity (W cm^−2^). Calibrations are checked at levels 10^−5^, 10^−7^ and 10^−9^ W cm^−2^, which correlated to light level values around 150, 110 and 70 respectively (see Figure S1).

The same procedure was applied to light profiles, also recorded by the TDLRs, to identify the depth of the most significant light inflection point for each dive. It is important to note consistent light-depth profile differences between the descent and ascent phase of each dive (Figure 1), owing to a time-response lag inherent in the light sensor that is greatest at low light levels (Wildlife Computers), as well as possible changes in water properties, surface irradiance and animal behaviour. We calculated the average depth of the most significant light inflection point for each dive to account for this bias. From the surface to the depth of the most significant light inflection point was considered the section of the water column that incorporated the bulk of phytoplankton.

**Figure 2:**
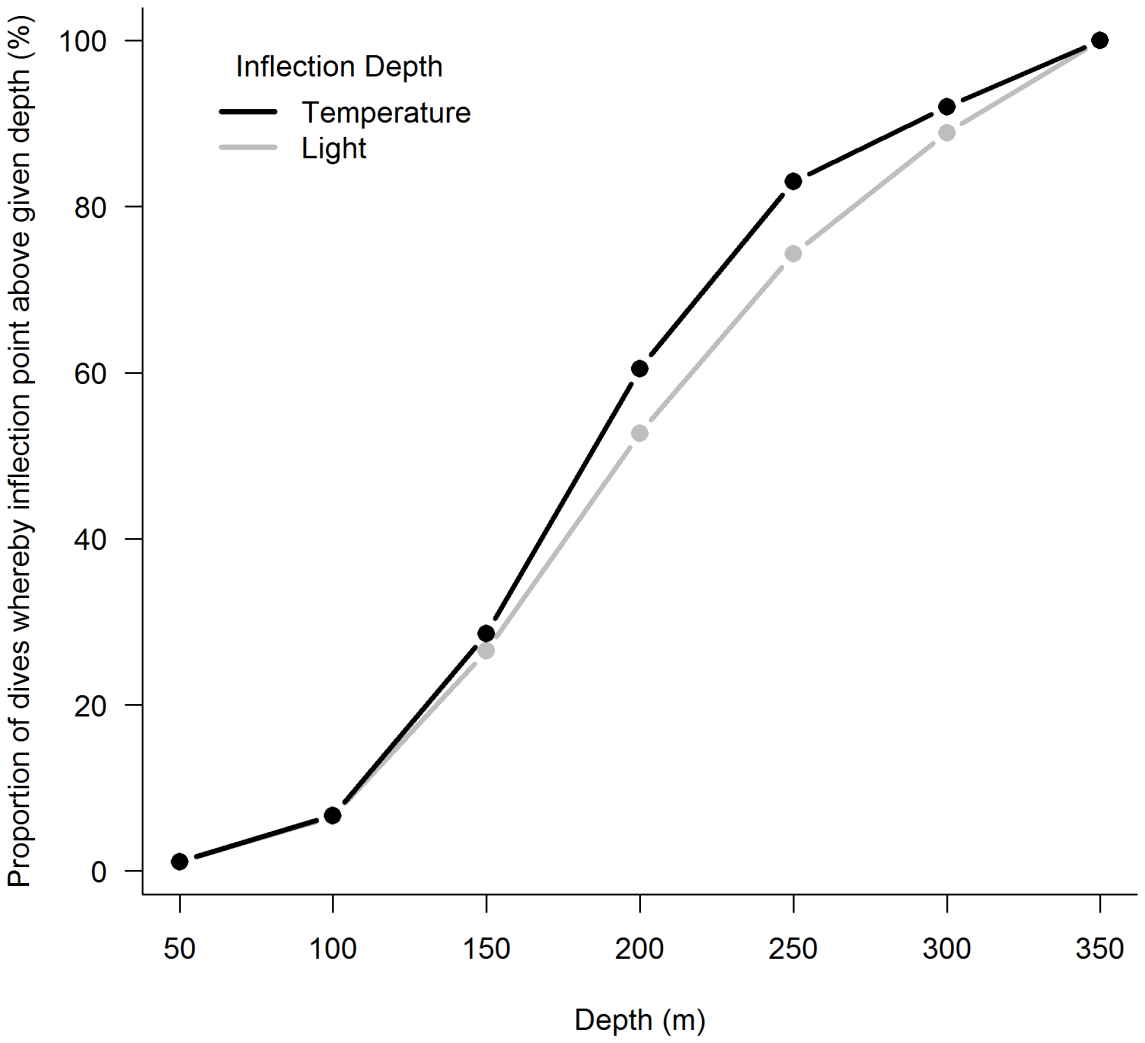
Proportion of dives whereby greatest temperature and light inflection points are above a given depth.

These analyses showed the proportion of dives with a given temperature and light inflection depth closely corresponds with each other (Figure 2). According to these results we conclude that the mixed layer depth and bulk of phytoplankton were frequently above 250 m (82.6% and 74.3% of dives respectively), and fewer dives encountered mixed layer depths or the bulk of phytoplankton exceeding 300 m (17.4% and 25.7%) (Figure 2).

#### Frontal zones

**Figure 3:**
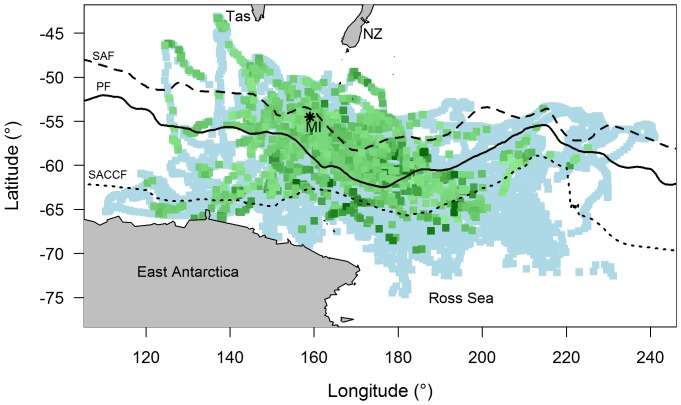
Noon locations for all seals with and without concurrent *chl-a*. Includes locations during post-moult and post-breeding foraging trips collectively (light blue), and of these, all that correspond with *chl-a* (green). Scale of *chl-a* values denoted by grading from light green (0.03 mg m^−3^) to dark green (2.48 mg m^−3^). Map shows the bottom of Tasmania (Tas) and New Zealand (NZ) and the coast of East Antarctica and Ross Sea (bottom). The black asterisk shows Macquarie Island (MI). Lines represent the historical mean positions of the Sub-Antarctic Front (SAF - dashed), Polar Front (PF - solid) and Southern Antarctic Circumpolar Current Front (SACCF - dotted).

Frontal structures in the Southern Ocean are sharp, horizontal gradients in water properties that mark the boundaries between different frontal zones (FZ) (Figure 3 – represented by historical mean front positions). The general position for each front can be marked using representative values of temperature and salinity at approximately 200 m depth. The FZ occupied by the seal was identified by water temperatures at 200 m depth (T_200_), as indicated by Park et al. [41] and Orsi et al. [42]. The sub-Antarctic Front (SAF) limit was defined by sub-surface values of 7°C, the Polar Front (PF) was defined by the northern limit of 2.8°C, and the Southern Antarctic Circumpolar Current Front (SACCF) was defined by a temperature of 1.6°C [43]. In this study we use these subsurface boundaries to distinguish between three major FZ: the Polar Frontal Zone (PFZ) was where seals encountered temperatures greater than 2.8°C; north of the southern Antarctic Circumpolar Current Front (SACCF-N) was where seals encountered temperatures between 2.8°C and 1.6°C; and south of the SACCF (SACCF-S) was where seals encountered temperatures below 1.6°C.

Temperature recorded for both the descent and ascent phase of individual dives were used and a temperature value at 200 m (T_200_) was derived for both the descent and ascent phase of each dive using a linear interpolation between the non-regular series of depths and temperature. Temperature values from the two phases were averaged. Because we only retained local noon light attenuation values for our analyses only local noon temperature estimates were used to calculate the mean daily noon T_200_. Each daily noon light level profile was assigned to a given FZ based on these mean daily noon T_200_ values (Figure S2).

### Light attenuation

By examining the mixed-layer depths and light-depth profiles encountered by the seals we determined that the bulk of phytoplankton was likely found in the top 250 m of the water column (see mixed layer section). Moreover, light levels recorded at depths of 300 m or more become unreliable as the light sensors reach their sensitivity limit. Consequently, integrated light attenuation between the surface and a depth of 250 m (*LA_250_*) was used as an index of phytoplankton in the water column (based on the assumptions outlined in our introduction).

To calculate *LA_250_*, light data were first interpolated linearly between the non-regular series of depths to estimate light levels at 250 m for each dive (*LL_250_*). We used light levels recorded for both the descent and ascent phase due to sensor and measurement error (see mixed layer section). The surface light level for each dive was estimated from the mean sub-surface light levels in the top 10 m of the water column at the end of the ascent phase (*LL_0_*) [12]. Light levels above the surface (indicated by the wet/dry sensor on the tag) were excluded from *LL_0_* estimates. For each dive, *LL_250_* was subtracted from *LL_0_* and divided by the depth (z) of *LL_250_* (*i.e.* 250 m) to calculate the *LA_250_* (m^−1^):
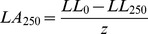
Only *LA_250_* values 1 h either side of local noon (1100–1300) were used in order to minimise variability in the ambient light field (see discussion in [10]). The interpolation of geo-locations was done to attempt best correspondence with noon dives and *chl-a* (see maps – Figure S2). This was based on the assumption that the seals' trajectory between consecutive locations was straight. Data recorded 1 h either side of local noon encompassed, on average, 4.3±1.3 light profiles per seal per day. Since we assume Southern Ocean waters are Case-1, attenuation will be dominated by phytoplankton (see introduction for details).

### Statistical analysis

As part of this study we aimed to demonstrate that *LA_250_* values (our phytoplankton index) are consistent with typical seasonal *chl-a* patterns. However, spatial error associated with positions derived by geo-location [17] impart uncertainty in the true position of the recorded light attenuation. If then compared to *chl-a* values, for which the spatial errors were considerably less, any resulting correlation may be subsequently weakened. We attempt to account for spatial bias in geo-location position errors by spatial averaging of the data into 1°×1° grid cells. Grid cells with less than 3 dive profiles were excluded from the analysis as these were likely to give unreliable estimates of the resulting mean *LA_250_* and mean *chl-a* per grid cell. Moreover, *chl-a* data were sparse at high latitudes (>64°S) and during winter months due to elevated cloud cover (Table 2). Conversely, the seal light data were sparse at low latitudes (<52°S) as few seals travelled north of this region (Table 2). Focal analysis was therefore based on data collected between 52°S and 64°S, and excluded winter months; for two reasons: (i) it is not possible to establish interaction effects when there are missing data in the dataset; and (ii) low data frequency may result in interaction effect bias.

**Table 2:** Frequency of daily locations for each season by 1° latitudinal bins.

Latitude (°S)	Season			
	Autumn	Spring	Summer	Winter
44	-	-	-	6
45	-	-	-	4
47	4	-	-	1
48	1	-	-	2
49	1	4	-	1
50	2	7	3	1
51	1	8	5	6
52	3	6	8	2
53	5	10	5	-
54	2	18	11	-
55	4	63	38	-
56	6	72	44	1
57	3	51	50	-
58	12	41	43	-
59	14	21	53	-
60	21	27	46	-
61	22	26	47	-
62	27	17	40	-
63	24	12	27	-
64	33	2	15	-
65	40	-	13	-
66	28	-	14	-
67	10	-	4	-
68	5	-	-	-
69	3	-	-	-
70	2	-	-	-

We investigated the relationship between *chl-a* and *LA_250_* aggregated at 1° resolution. We used the mixed effect model (*nlme*) package in R [44] to assess this relationship with and without the random intercept term and slope effect to determine whether individual seals were contributing to the model fit. Season and latitude (and their interaction terms) were included in our analysis because of their likely effect on phytoplankton abundance in the water column (for details see discussion). Season was divided according to the austral seasonal cycle: summer (Dec–Feb); autumn (Mar–May); winter (Jun–Aug); and spring (Sep–Nov). Because FZ is largely influenced by latitude in the Southern Ocean (e.g., [45], [46]) we expect the inclusion of FZ and latitude in our mixed model to have a confounding effect on *chl-a* distribution. For that reason we assessed the inclusion of each of these effects in our mixed model relative to each other and found that latitude was more useful for the purpose of this study (Table S1). We therefore tested the individual fixed effects (including *LA_250_*, season, latitude and their interactions) by sequentially removing non-significant terms from the model according to Zuur et al. [47]. In all cases, models were ranked via Akaike Information Criterion [48], the most parsimonious model having the lowest AIC value. Model selection was carried out using Maximum Likelihood (ML) estimation. In addition, we used *F* and *t* statistics to examine the significance of individual fixed effects. The final model is presented using restricted maximum likelihood (REML) methods. Both *chl-a* and *LA_250_* values were log-transformed to ensure a normal distribution.

## Results

We used data from entire foraging trips for 67 (75%) of the 89 deployments (31 post-breeding/36 post-moult trips). Twenty one trips were excluded due to light sensor failure at some point during the time at sea. Data for one seal were also omitted due to unrealistic track estimates (*i.e.* the track passed over land). Data were obtained over 1561 days from 22 Oct 1999 through to 8 Oct 2005. A total of 31614 light profiles at 9552 noon locations were recorded during this period (Table 3)). There were 7212 noon locations available that included 3 or more light profiles (*i.e. LA_250_* values) and did not coincide with heavy sea-ice, of which only 1461 noon locations coincided with *chl-a* values (20.3%) (Table 3, Figure 3). This showed approximately one-fifth of seal locations (with daily *LA_250_* values) coincided with *chl-a* values. Filtered data (*i.e.* included 3 or more light profiles, did not coincide with heavy sea-ice) were then gridded into 3940 1°×1° cells for model analysis, of which only 1066 cells (25.1%) corresponded with gridded *chl-a* data (Table 3). Each cell incorporated 1.26±0.02 locations (4.77±0.09 light profiles). Seals travelled either to the sea ice zone in the north of the Ross Sea and off the coast of East Antarctica, or to the shelf break of East Antarctica (Figure 3). Most seals travelled to areas south of the SACCF.

**Table 3:** Data summary for each deployment (*i.e.* trip) by year: number of seals (*n*); total light profiles and locations at noon and concurrent chl-a; filtered† light profiles and locations at noon and concurrent chl-a; number of 1° grid cell locations and concurrent *chl-a*.

			Total			Filtered†			Grid Cells	
Year	Trip	Seals (n)	Light profiles	Locations	Concurrent chl-a	Light profiles	Locations	Concurrent chl-a	Locations	Concurrent chl-a
1999	PB	14	4319	984	307	4096	939	306	514	207
2000	PB	10	2954	713	167	2805	681	165	365	115
2001	PB	-	-	-	-	-	-	-	-	-
2002	PB	-	-	-	-	-	-	-	-	-
2004	PB	7	1733	433	180	1644	415	177	255	129
2005	PB	-	-	-	-	-	-	-	-	-
		*31*	*9006*	*2130*	*654*	*8545*	*2035*	*648*	*1134*	*451*
1999	PM	-	-	-	-	-	-	-	-	-
2000	PM	6	4120	1328	113	3385	1051	111	544	89
2001	PM	5	3091	951	93	2659	808	85	433	69
2002	PM	10	6199	2001	269	4241	1262	254	689	188
2004	PM	11	6832	2292	297	5034	1632	283	892	205
2005	PM	4	2366	850	81	1312	424	80	248	64
		*36*	*22608*	*7422*	*853*	*16631*	*5177*	*813*	*2806*	*615*
	**Total**	**67**	**31614**	**9552**	**1507**	**25176**	**7212**	**1461**	**3940**	**1066**
	**%**	**-**	**-**	**-**	**15.8**		**-**	**20.3**	**-**	**27.1**

† locations with >3 light profiles that do not coincide with heavy sea-ice.

### Relationship between light attenuation and *chl-a*

**Figure 4:**
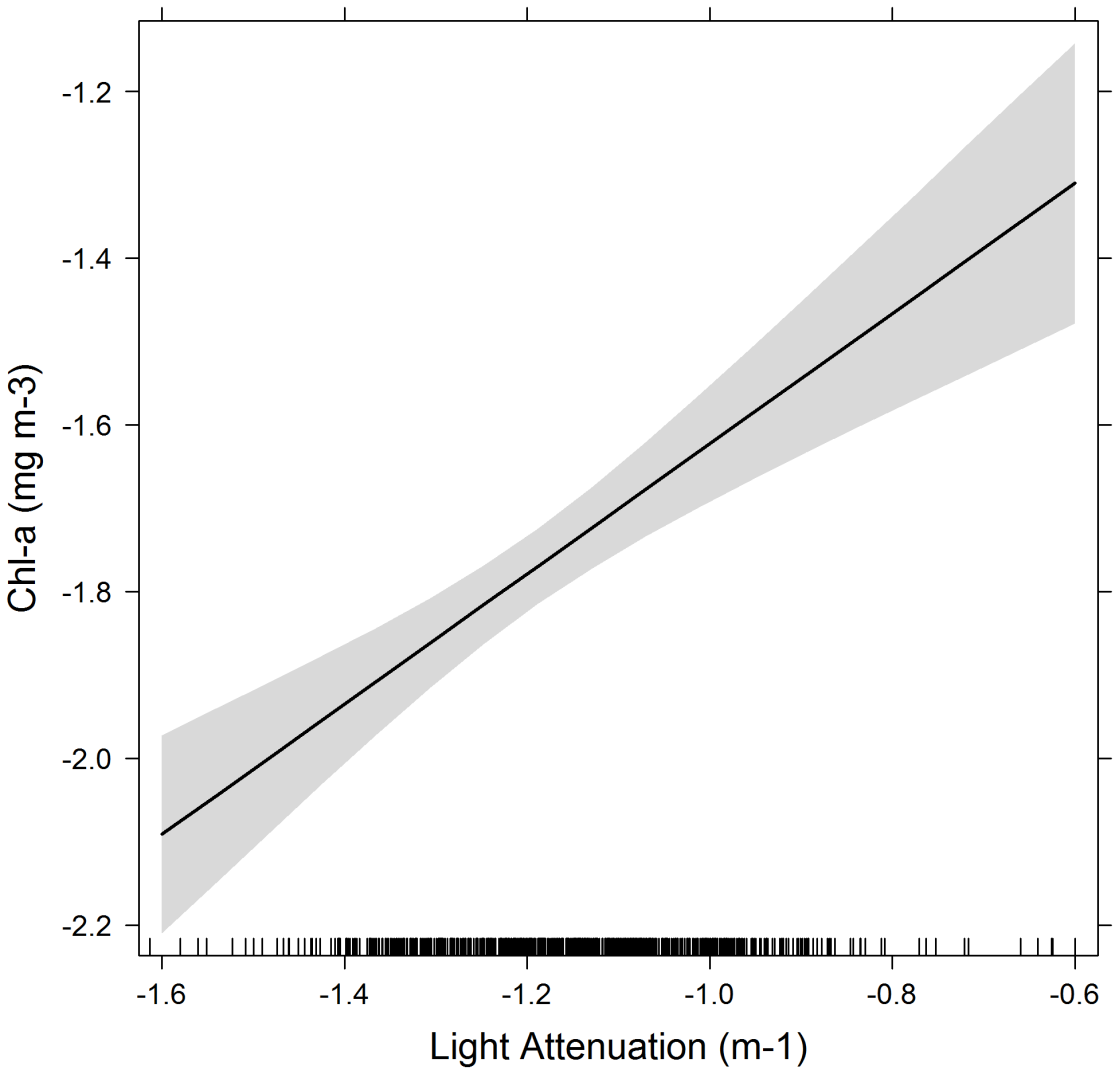
Relationship between *chl-a* and light attenuation (LA_250_) from our mixed model. Shaded area indicates the confidence level. Both axes are log transformed.

**Figure 5:**
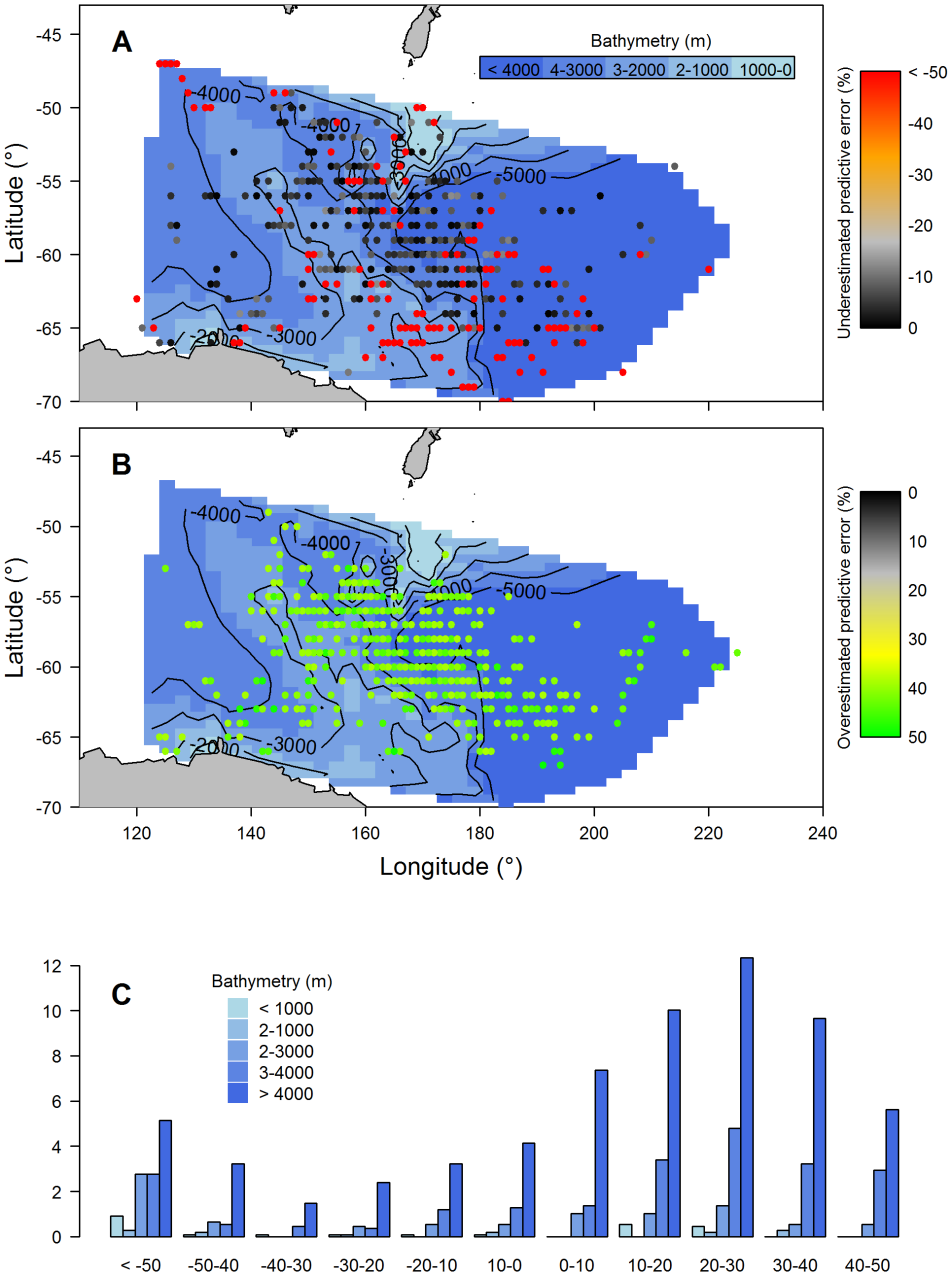
Spatial distribution of predictive chlorophyll-a error from our mixed model. Plots show locations associated with (A) underestimated predictive error (%), (B) overestimated predictive error (%), and (C) proportion (%) of locations with associated predictive error (%) in relation to bathymetric bands.

The best model relating *LA_250_* to *chl-a* included individual variability (random intercept term *seal*), the random slope term (*LA_250_*); most parsimonious model included fixed effects *LA_250_*, season, latitude, and the 2-way interaction terms *LA_250_*: latitude and latitude: season (Table 4). The *LA_250_* was positively related to *chl-a* (estimated coefficient = 0.76±0.13, p<0.0001, Table 5, Figure 4). Predicted *chl-a* values from our model show no obvious latitudinal or longitudinal error pattern over the study region (Figure 5A, B). However, results indicated that predicted *chl-a* values largely overestimated *chl-a* by 10–30%, particularly over water depths greater than 4000 m, those most frequented by seals (Figure 5C).

**Table 4:** Ranked mixed models.

Candidate models	*df*	AIC	ΔAIC	logLik
**LA_250_+lat+S+LA_250_: lat+S: lat**	**12**	**547.8**	**0**	**−261.9**
LA_250_+lat+S+S: lat	11	550.7	2.9	−264.3
LA_250_+lat+S+LA_250_: S+LA_250_: lat+S: lat	14	551.7	4	−261.9
LA_250_+lat+S+LA_250_: S+S: lat	13	553.9	6.2	−264
LA_250_+lat+S+LA_250_: S+LA_250_: lat+S: lat+LA_250_: S: lat	16	554.2	6.5	−261.1
LA_250_+lat+S+LA_250_: lat	10	554.5	6.7	−267.2
LA_250_+lat+S	9	557.1	9.3	−269.5
LA_250_+lat+S+LA_250_: S+LA_250_: lat	12	558.5	10.7	−267.2
LA_250_+lat+S+LA_250_: S	11	560.3	12.5	−269.1
LA_250_+S	8	568.1	20.4	−276.1
LA_250_	6	662	114.2	−325
LA_250_+lat	7	662.7	115	−324.4
S+lat	6	664.8	117.1	−326.4
S	5	685.2	137.5	−337.6
lat	4	787.1	239.3	−389.5
∼1	3	794.5	246.7	−394.2

The *chl-a* explained by light attenuation at 250 m (LA_250_), season (S) and latitude (lat) (*n* = 67 seals). Mixed models are ranked by decreasing Akaike's Information Criterion (AIC) and change in AIC (*Δ*AIC). The most parsimonious model is in bold.

**Table 5:** Results from the most parsimonious mixed model: relating *chl-a* to integrated light attenuation in the top 250 m of the water column (*LA_250_*), latitude and season and their significant interactions.

	Coefficient ± SE	Coefficient p
**LA_250_**	**0.76±0.13**	**<0.0001**
**Season (Summer)**	**0.33±0.04**	**<0.0001**
**Season (Spring)**	**0.21±0.04**	**<0.0001**
Latitude	0.06±0.03	0.0654
**LA_250_: Latitude**	**0.06±0.03**	**0.0264**
**Season (Summer): Latitude**	**0.03±0.01**	**0.0071**
**Season (Spring): Latitude**	**0.03±0.01**	**0.0040**

Term coefficients are presents ± SE and *p*-values for each coefficient are also shown. Significant terms (*p*<0.05) are denoted by bold characters. For the season variable that was a factor in the model, coefficients are given in reference to autumn.

### Distribution of light-based *chl-a* estimates

**Figure 6:**
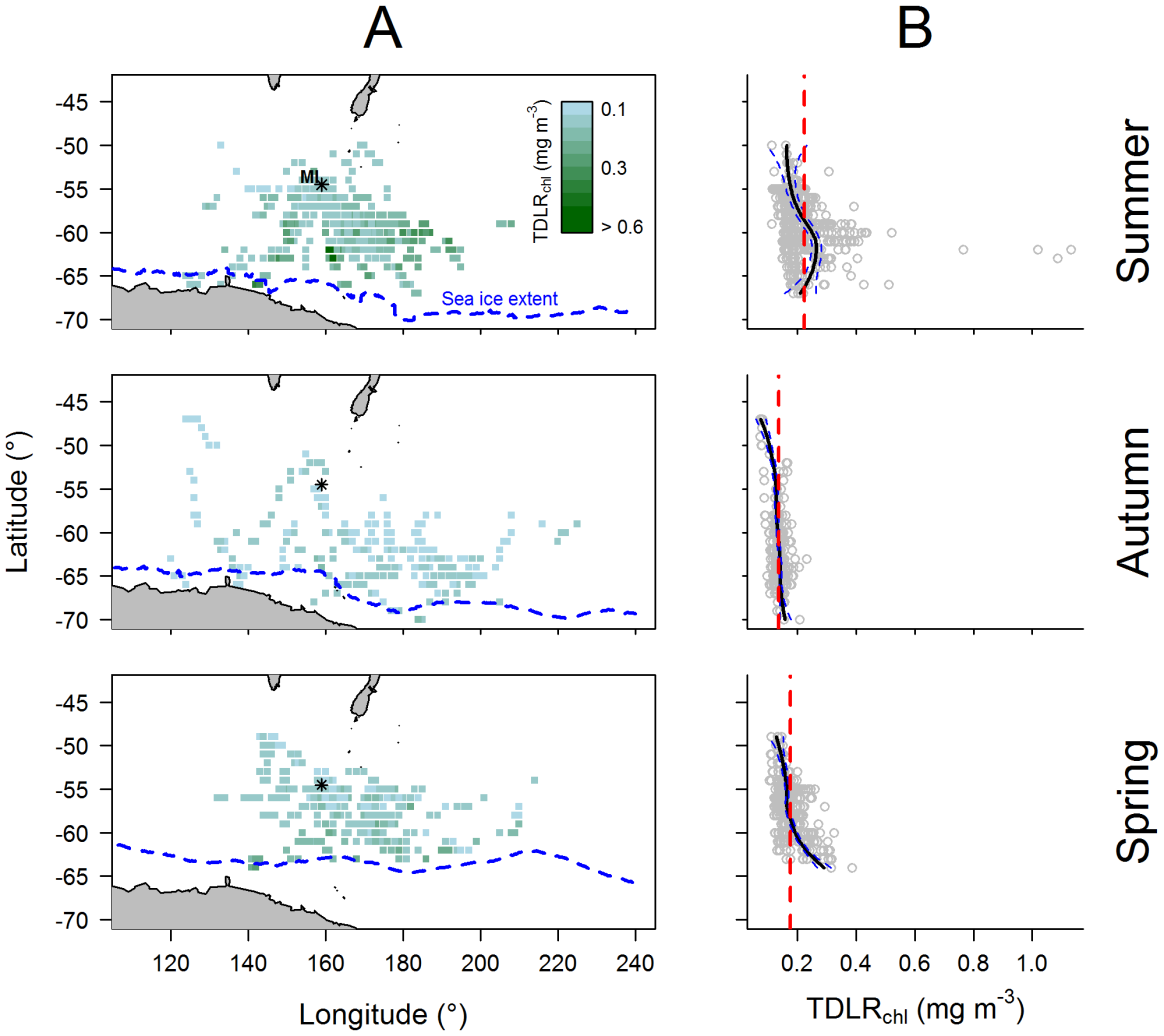
(A) Seasonal spatial distribution and (B) latitudinal patterns of TDLR_chl_† from the final mixed model. Each map shows the bottom of Tasmania and New Zealand (top), the coast of East Antarctica and Ross Sea (bottom), and the sea ice extent (blue dashed line). The black asterisk shows Macquarie Island. For each corresponding plot (B) the black line represents a loess fit and blue dashed lines represent the 95% confidence level, and the vertical red dashed line represents the mean TDLR_chl_. †Light-based *chl-a* estimates from our final mixed model collected by TDLRs.

Fitted values from the mixed model results (*i.e.* phytoplankton index) were used to calculate the spatial distribution of light-based *chl-a* collected by TDLRs (hereafter TDLR_chl_) encountered by the focal seals (Figure 6A), revealing different seasonal patterns in relation to latitude (Figure 6B). During summer months, seals encountered generally higher TDLR_chl_ compared to other times of the year, particularly at latitudes between 60°S and 65°S, south-east of Macquarie Island (north of the Ross Sea). Conversely, seals encountered uniformly low TDLR_chl_ across latitudes during autumn. In spring, TDLR_chl_ encountered by seals were marginally greater (Figure 6B), and levels gradually elevated toward the mean spring-time sea ice extent.

**Figure 7:**
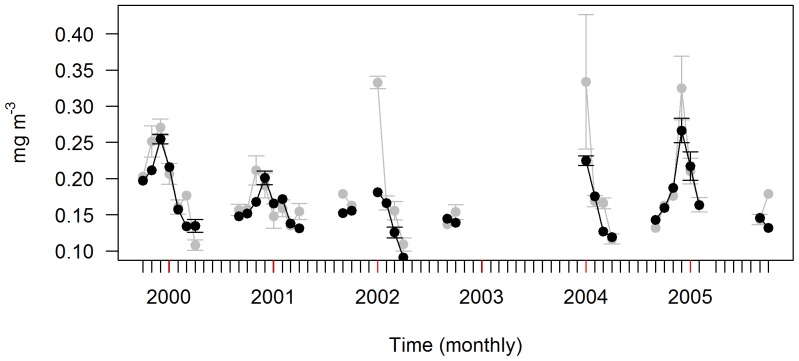
Mean inter-annual cycles. The *chl-a* (grey) and light-based *chl-a* estimates from our final mixed model collected by TDLRs (TDLR_chl_) – black) mean inter-annual cycles within a 55°S to 60°S latitudinal band of the study site (*i.e.* where seal density is highest – see Table 3). Values include standard error bars. Red ticks on the x-axis represent January of each year. N.B. mean inter-annual trends are incomplete because study lacked PB deployments for 2001, 2002 and 2003, and PM deployments for 2003 (see Table 1). Furthermore, mixed model analysis excluded winter months (see Results).

These same fitted values were also used to calculate inter-annual TDLR_chl_ variability and compared with *chl-a* within the 55–65°S latitudinal band (Figure 7). Mean monthly TDLR_chl_ agreed well with *chl-a* inter-annual variability, despite large differences for January 2002 and 2004, and to a lesser extent, December 2005. These large differences correspond well with the few available data (Figure 7).

### Light verses *chl-a* data coverage

**Figure 8:**
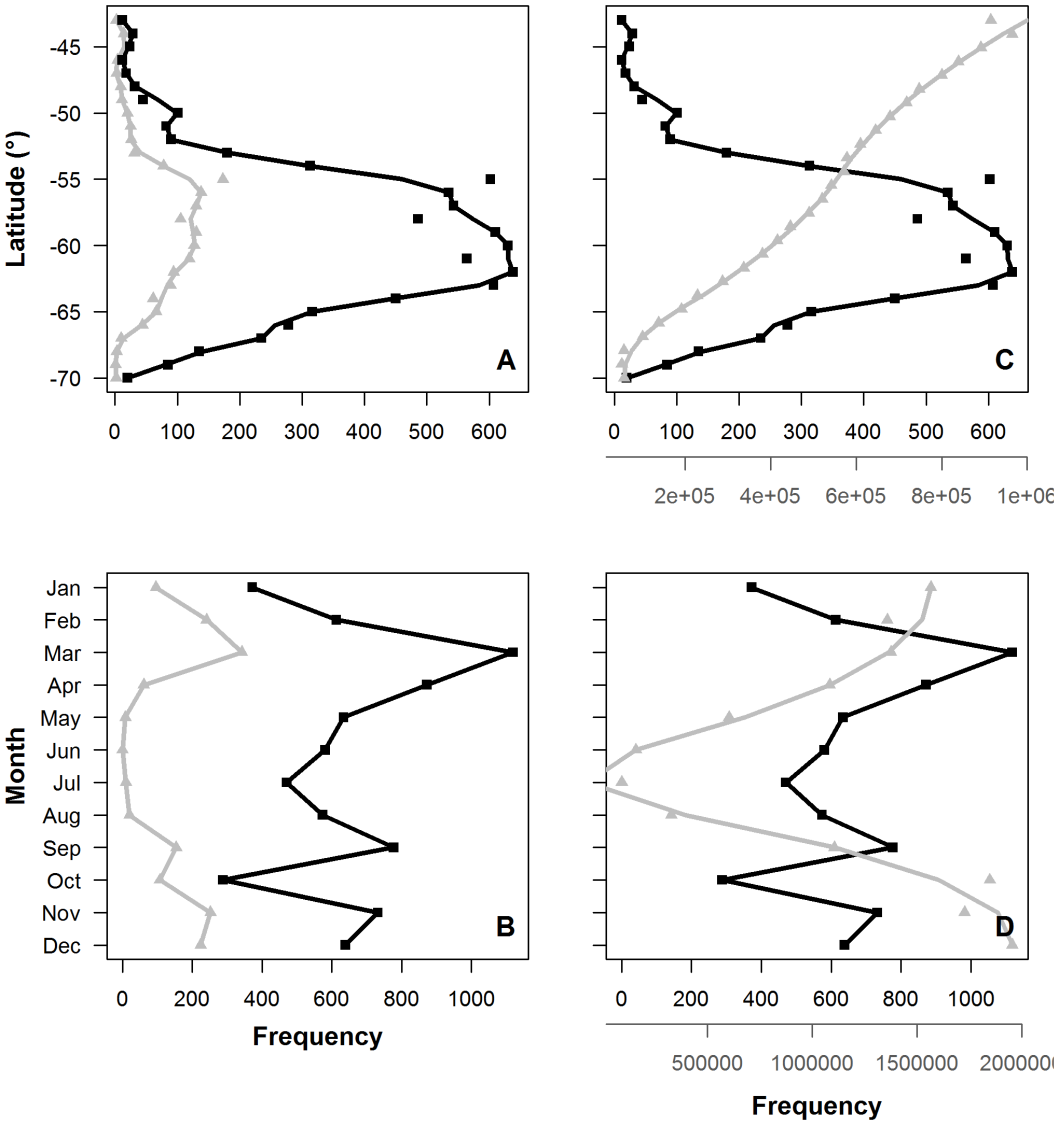
Data frequency coverage of chl-a* and TDRL_chl_†: data coverage of the study region is shown by (**A**) latitude (at 1° increments) and (**B**) months (between 1999 and 2005). Black represents TDRL_chl_ coverage and grey represents *chl-a* coverage. Lines represent a loess fit. * *chl-a* data. †Light-based phytoplankton index calculated from TDLR data.

The TDLR_chl_ data sets provided more information than the *chl-a* data that corresponded with seal locations (hereafter corresponding *chl-a*), but both followed similar spatial and temporal trends (Figure 8A and Figure 8B respectively). However, coverage of TDLR_chl_ and the overall *chl-a* available within the focal study region (hereafter overall *chl-a*) each followed different spatial and temporal trends (Figure 8C and Figure 8D respectively).

Spatial coverage of TDLR_chl_ and corresponding *chl-a* peaked at latitudes between 55°S and 64°S; however peak corresponding *chl-a* coverage was considerably less than TDLR_chl_ coverage (Figure 8A). In general, peak TDLR_chl_ coverage increased with latitude up to 64°S only to drop with increasing proximity to the Antarctic Continent. However, the extent of TDLR_chl_ coverage was still considerable at latitudes as high as 67°S. Conversely, peak corresponding *chl-a* data coverage steadily decreased from 56°S, becoming virtually negligible at 66°S. Overall, *chl-a* data coverage was greatest at 44°S, but was inversely related to latitude; virtually negligible at latitudes greater than ∼67°S (Figure 8C).

Temporal coverage of TDLR_chl_ and corresponding *chl-a* data peaked twice over a 12-month period; the largest peak during March, the other during spring with the exception of a sharp drop of coverage in October (Figure 8B). However, the two peaks in TDLR_chl_ coverage were considerably greater than that of corresponding *chl-a* data (Figure 8B). Less coverage of TDLR_chl_ and corresponding *chl-a* data was evident at the beginning of summer, during winter and in October. Specifically, minimal coverage of TDLR_chl_ occurred in October during the breeding season, although still maintained moderate-to-low coverage at this time compared to virtually nil coverage of corresponding *chl-a* data throughout winter. Overall *chl-a* data coverage was poor during the winter months, particularly in July when overall *chl-a* data coverage was completely unavailable (Figure 8D).

## Discussion

This is the first multi-year dataset (67 elephant seals) used to provide a light-based index of phytoplankton density that is concurrent with a marine animal's entire foraging trip over multiple seasons. Of the 3940 *LA_250_* gridded cells recorded over 5 years, only 25.1% cells coincided with *chl-a* measurements demonstrating the deficiency of remotely sensed data sources concurrent with animal behaviour. Model output also revealed that seasonal trends detected by our phytoplankton index were in agreement with data collected by remote sensing. It demonstrates how our phytoplankton index is consistent with near-surface *chl-a* values in the Southern Ocean, and that phytoplankton changes at depth generally reflect near-surface primary producer conditions. This opens the way for the use of simple light data as a bio-optical index for phytoplankton in the Southern Ocean that is concurrent with animal at-sea behaviour.

### Relationship between water column light and *chl-a*

The Southern Ocean is characterised by Case I waters, where phytoplankton organisms are the most optically significant components of the water column [22]. It is therefore likely that bio-optical differences detected by TDLR light sensors at depth are representative of plankton densities. Our results show that *chl-a* (derived from satellite images) is significantly related to our phytoplankton index estimated from the integrated light attenuation recorded by TDLRs in the top 250 m of the water column. Light at 250 m generally coincides with the limit of the euphotic zone, so all photosynthetic organisms in the water column influence light attenuation at this depth. In general, there is a good relationship between *chl-a* concentration within the top 30 m (as detected by satellite images) and chlorophyll-a integrated over the entire euphotic zone [11]. Nevertheless, because the density of phytoplankton particulates is particularly low in the Southern Ocean [1], [49] perhaps the compounding effect of phytoplankton cells on *LA_250_* enables TDLR sensors to detect differences that correlate well with *chl-a*. Preliminary analysis in this study suggests that most phytoplankton, which is retained within the mixed layer, is often found in the top 250 m of the water column. Furthermore, because phytoplankton is virtually negligible below the euphotic zone [50] no further information is likely to be gained by considering depths greater than 250 m.

We only used data from a 2 h period around the local noon to reduce the influence of ambient light field variability, thereby improving the accuracy and reducing the variability of light attenuation between dives (for details see [10]). However, solar elevation angle at local noon is affected by latitude and time of year (*i.e.* season), invariably altering light penetration at depth, and ultimately, light attenuation at 250 m. This would consistently affect the relationship between *chl-a* and *LA_250_* as seals travel extensively across the Southern Ocean. We suggest that the interactions between *LA_250_* and latitude, as well as latitude and season, were retained in our final model to account for changes to the solar elevation angle at local noon. Variability of light attenuation in the water column is also due partly to differences in optical properties between phytoplankton species [25], [51]. Different phytoplankton groups (based on their bio-optical characteristics) can be highly influenced by latitude (e.g., haptophytes and diatoms are found mostly in high latitudes [52]) and season (e.g., diatoms blooms dominate during spring and summer [52]). Moreover, different FZ can influence distribution of phytoplankton groups [53]–[55], and therefore, light attenuation variability, although we suggest that latitude can account for this effect in the Southern Ocean. It is possible, however, that distinct phytoplankton assemblages are not closely associated with our defined FZs. Perhaps a better understanding of FZ and their associated phytoplankton assemblages may show, in fact, that FZ is a useful contributing predictor to our light-based index of phytoplankton distribution. Finally, one of the reasons for using *LA_250_* (*i.e.* relative decrease in irradiance) is that it normalises out small variations in sensor sensitivity or calibration. Nonetheless, seal was included as a random term in mixed model analyses despite Wildlife Computers checking light sensor calibrations (see Figure S1). We expect light values still vary between individual seals and potentially influence the relationship between light and *chl-a* if not accounted for.

These findings show the value of using existing datasets collected from animal-borne light sensors to calculate an index for phytoplankton density in the water column. Indeed, our results revealed that seasonal trends detected by our phytoplankton index were in agreement with data collected by remote sensing. This is despite temporal and spatial accuracy issues associated with both *chl-a* and *LA_250_* data that were a potential source of persistent error in our analysis. First, typically dense cloud cover in the Southern Ocean (particularly during winter and at high latitudes) required use of 8-day composite SeaWiFS data (rather than 1-day) to improve data coverage, thereby compromising temporal resolution. Second, we expect spatial error inherent in our geo-location estimates (see methods) to result in spatial mismatch between *LA_250_* and *chl-a*. Analyses were performed at 1° degree resolution to minimise location error bias. Third, it is possible that body position of the diving seals affects detection of irradiance by the light sensor. Indeed Sala et al. [56] have shown how body roll is incorporated into typical diving bouts throughout a seal's entire foraging trip, although we see little evidence of body position affecting light profiles (for examples refer to Figure 1) and expect error due to roll to be minimal. Although these issues may exist in our analysis we were still able to show how our phytoplankton index revealed seasonal trends consistent with data from *chl-a*.

It is likely that much of the discrepancy in our model between *chl-a* and *LA_250_* largely originates from our data sources. Satellites do not provide a direct measure of *chl-a* and instead measure radiance and use empirically derived algorithms to estimate values. The SeaWiFS algorithm used for estimating *chl-a* tends to underestimate values in the Southern Ocean [57], [58]. Surface prey aggregation may also contribute to the overestimation of *chl-a* detected during spring when zooplankton in particular become more abundant [59]. However, it is also possible that these prey aggregations could consistently coincide with elevated *chl-a* and therefore still correlate well with surface (or shallow subsurface) *chl-a* in any case. Nonetheless, we would expect that subsurface biology accounts for some of the discrepancy between our phytoplankton index and *chl-a*. Prey aggregations (*e.g.* zooplankton, fish), for instance, can affect light attenuation [10], [12], which become increasingly likely with depth. Moreover, deep chlorophyll-a maxima (DCM) can be more than 30% that of surface values in some regions [11] and may cause further decoupling of *chl-a* and *LA_250_*. Holm-Hansen et al. [60] showed that DCMs are located predominately over the deep ocean basins, regions regularly frequented by the focal elephant seals. Indeed, model predictions were more likely to overestimate *chl-a* by 10–30% when light was recorded over bathymetry greater than 4000 m (Figure 5). Our light-based phytoplankton index may therefore be useful for estimating total phytoplankton densities in the water column, rather than only providing near-surface *chl-a* information where seals dive.

### Ecological significance

The light-based phytoplankton index from our model (hereafter phytoplankton index) produced seasonal patterns typical of *chl-a* distribution in the Southern Ocean south of Australia and New Zealand [61]. Summer values were consistent with Sokolov and Rintoul [61] that showed relatively high phytoplankton south of the Antarctic Circumpolar Current, and where the Polar Front interacted with the Mid-Ocean Ridge (*i.e.* regions between 60°S and 65°S). Particulate density estimates also show typical seasonal patterns that are in agreement with *chl-a* values for the same region (Sokolov and Rintoul [61]: low *chl-a* across the entire Southern Ocean leading into austral winter and a rise in early spring in the vicinity of the sea ice extent. It is important to consider, however, that locations visited by the seals may result in biased phytoplankton distributional trends. For example, in spring, seals may target slightly elevated phytoplankton patches at high latitudes and therefore not sample the relatively low phytoplankton densities of surrounding areas. We did, however, show that an inter-annual trend in mean monthly phytoplankton estimates corresponded well with *chl-a* within a latitudinal band most frequented by the focal seals; further validating that light-based estimates are detecting biological activity.

This study also shows that TDLRs record data in areas where satellite coverage is limited or completely absent. Specifically, satellite coverage is poor at high latitudes and during winter where cloud and ice coverage is more prevalent. This lack of data potentially limits our understanding of resource distribution in the Southern Ocean in relation to seal movement and their foraging behaviour [5]. Electronic tags deployed on animals have already been used to collect *in situ* temperature and salinity data along its track to improve our understanding of habitat utilisation [62]. Using light to estimate relative phytoplankton distribution may prove a useful covariate recorded simultaneously with elephant seal behaviour in future studies, particularly at the large scale and where *chl-a* data is sparse as described here. Indeed, our light-based phytoplankton index recorded at depth could be more relevant to a deep diving apex predator rather than *chl-a* data taken at the near-surface, although this is beyond the scope of this study. Regardless, these light data are already widely available, for a range of marine species, as light is traditionally recorded for estimating geo-location. This provides an opportunity to augment the application of light data in this study with data collected by multiple species.

Phytoplankton blooms typically support high zooplankton densities [59], [63], [64], and this in turn provides an important food resource for pelagic fish and higher predators. Traditionally, *chl-a* data has been the primary source of resource information in the marine environment, but are often limited by cloud cover at high latitudes and lack information at depth. Studies have often not found any significant relationship between *chl-a* and top predators foraging movements (e.g., [65]), unless at large scales, where general associations are apparent (e.g., [66]). In some instances foraging behaviour has even been shown to be inversely related to *chl-a* (e.g., [5]). However such studies cite either a lack of *chl-a* data [5], [6], limited satellite resolution [6], [67] or “downstream” effects decoupling phytoplankton from its physical conditions of origin [67] as possible explanations. It is therefore crucial that concurrent data is used where possible in efforts to model and understand trophic linkages in Southern Ocean ecosystems. Recording animal behaviour and light data simultaneously may enable researchers to help improve the predictive capacity of ecological models. Light data may provide important biological context in regions of the Southern Ocean and during specific months of the year that are historically poorly understood. Tag configuration that incorporates both fluorometer and light sensors could improve our ability to disseminate between phytoplankton and zooplankton distribution in the 3D marine environment. These data could give new insight into the biology of foraging habitat and/or oceanographic structures (e.g. upwelling eddies, ocean fronts) visited by tagged animals by providing information on resource distribution.

## Supporting Information

Figure S1 **The relationship between relative light level and blue light intensity (W cm^−2^) for a typical tag.** Calibrations are checked by Wildlife Computers at levels 10^−5^, 10^−7^ and 10^−9^ W cm^−2^, which correlates to light level values around 150, 110 and 70 respectively. Furthermore, these light level values roughly equate to specific daylight conditions ranging from full sunlight to overcast night. Source: Wildlife Computers, USA.
(TIF)

Figure S2 **Locations of each annual deployment cohort.** Each location is assigned to one of three frontal zones: Polar Frontal Zone (PFZ – orange); north of the southern Antarctic Circumpolar Current (SACCF-N - green); south of the Southern Antarctic Circumpolar Current Front (SACCF-S - blue). Maps show the bottom of Tasmania (Tas) and New Zealand (NZ, top) and the coast of East Antarctica and Ross Sea (bottom). The black asterisks show Macquarie Island (MI).
(TIF)

Table S1 **Ranked mixed models at 1° resolution.** Satellite-derived chlorophyll (*chl-a*) explained by integrated light attenuation above 250 m (LA_250_), season, latitude and frontal zone (FZ) (*n* = 67 seals)^†^. Mixed models are ranked by decreasing Akaike's Information Criterion (AIC) and change in AIC (*Δ*AIC) [41]; the most parsimonious model having the lowest AIC.
(DOCX)
