# Supplementary figures and images for: Estimating Trans-Seasonal Variability in Water Column Biomass for a Highly Migratory, Deep Diving Predator
Source: PLoS One. 2014 Nov 26;9(11):e113171. doi: 10.1371/journal.pone.0113171 (PMC4245103; doi:10.1371/journal.pone.0113171)

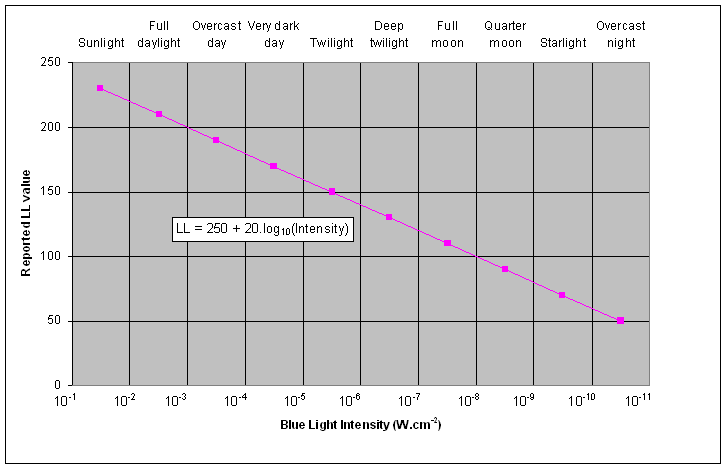

Supplement: Figure S1 — The relationship between relative light level and blue light intensity (W cm−2) for a typical tag. Calibrations are checked by Wildlife Computers at levels 10−5, 10−7 and 10−9 W cm−2, which correlates to light level values around 150, 110 and 70 respectively. Furthermore, these light level values roughly equate to specific daylight conditions ranging from full sunlight to overcast night. Source: Wildlife Computers, USA. (TIF) [file pone.0113171.s001.tif]

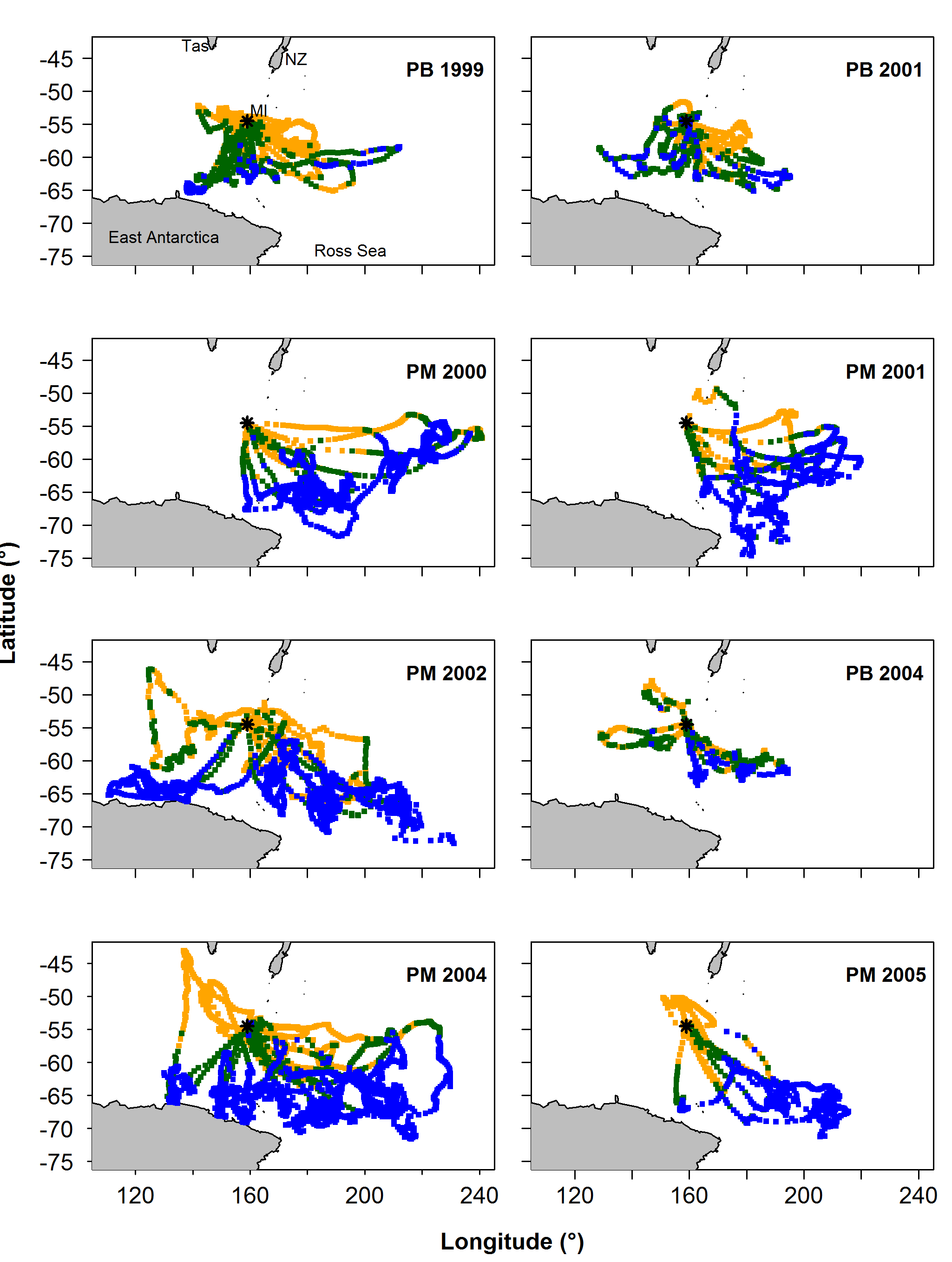

Supplement: Figure S2 — Locations of each annual deployment cohort. Each location is assigned to one of three frontal zones: Polar Frontal Zone (PFZ – orange); north of the southern Antarctic Circumpolar Current (SACCF-N - green); south of the Southern Antarctic Circumpolar Current Front (SACCF-S - blue). Maps show the bottom of Tasmania (Tas) and New Zealand (NZ, top) and the coast of East Antarctica and Ross Sea (bottom). The black asterisks show Macquarie Island (MI). (TIF) [file pone.0113171.s002.tif]
