# Supplementary material for: Estimating Trans-Seasonal Variability in Water Column Biomass for a Highly Migratory, Deep Diving Predator
Source: PLoS One. 2014 Nov 26;9(11):e113171. doi: 10.1371/journal.pone.0113171 (PMC4245103; doi:10.1371/journal.pone.0113171)
Supplement: Table S1 — Ranked mixed models at 1° resolution. Satellite-derived chlorophyll (chl-a) explained by integrated light attenuation above 250 m (LA250), season, latitude and frontal zone (FZ) (n = 67 seals)†. Mixed models are ranked by decreasing Akaike's Information Criterion (AIC) and change in AIC (ΔAIC) [41]; the most parsimonious model having the lowest AIC. (DOCX) [file pone.0113171.s003.docx]

| **Model*** | **df** | **AIC** | $\Delta$**AIC** | **BIC** | **logLik** |
| --- | --- | --- | --- | --- | --- |
| LA_250_ * season * latitude | 16 | 697.0 | 0 | 777.8 | -332.5 |
| LA_250_ * season * FZ | 22 | 740.8 | 43.7 | 851.8 | -348.4 |

**Table S1. Ranked mixed models at 1° resolution.** Satellite-derived chlorophyll (*chl-a*) explained by integrated light attenuation above 250 m (LA_250_), season, latitude and frontal zone (FZ) (*n*=67 seals)^†^. Mixed models are ranked by decreasing Akaike’s Information Criterion (AIC) and change in AIC ($\Delta$AIC) [41]; the most parsimonious model having the lowest AIC.

^†^season (levels: summer, autumn and spring); latitude (between 52°S and 64°S at 1° intervals); FZ (levels: SAFZ, PFZ and sSAACF).
